# Blood–brain barrier permeability to enrofloxacin in Pengze crucian carp (*Carassius auratus var*. Pengze)

**DOI:** 10.1002/vms3.918

**Published:** 2022-08-29

**Authors:** Fan Zhang, Runping Wang, Jianzhen Huang, Haixin Zhang, Long Wang, Zhiwei Zhong, Jiming Ruan, Huazhong Liu

**Affiliations:** ^1^ Department of Aquaculture College of Animal Science & Technology Jiangxi Agricultural University Nanchang P.R. China; ^2^ Jiangxi Fisheries Research Institute Nanchang P.R. China; ^3^ Bureau of Agriculture and Rural Affairs of Pengze County Pengze P.R. China; ^4^ College of Chemistry & Environmental Science Guangdong Ocean University Zhanjiang P.R. China

**Keywords:** blood–brain barrier, drug residues, enrofloxacin, Pengze crucian carp

## Abstract

**Background:**

Enrofloxacin (ENR) is a kind of quinolone antibiotic that is most widely used antimicrobials in veterinary practice, and possesses both a broad spectrum antimicrobial activity against a range of bacteria and adverse effects towards plants and animals.

**Objectives:**

This study was conducted to explore the permeability of blood–brain barrier (BBB) to ENR and brain injury based on crucian carp orally administrated with high dose of ENR.

**Methods:**

Juvenile Pengze crucian carp were treated with half lethal dose (LD_50_) or safe dose (SD_50_) of ENR. BBB permeability was determined by evaluating ENR contents detected by HPLC and evens blue contents estimated by confocal laser scanning microscope. Brain damage was evaluated by measuring protein and mRNA contents of related molecules with western blotting and qPCR.

**Results:**

Data indicated that ENR destroyed BBB structure of crucian carp and enhanced permeability of the biological barrier, resulting in more ENR crossed BBB and induced brain damage of crucian carp.

**Conclusions:**

This data indicated that ENR can induce brain damage of crucian carp through destroying BBB structure and enhancing permeability.

## Graphical abstract

(1) High dose of ENR damages BBB structure of crucian carp and increases BBB permeability.

(2) ENR induces brain damage of crucian carp that can be indicated by S100B and GFAP protein contents in serum.

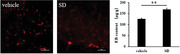

## 1 INTRODUCTION

Blood–brain barrier (BBB) is considered to have a highly protective effect on CNS of organisms (Pardridge, 1999). In animal brain, BBB is mainly composed of brain microvascular endothelial cells (BMECs), astrocytes (ACs), pericytes (PCs) and tight junctions (TJs) of cells or protein skeletons (Kadry et al., 2020). BBB has almost 100% barrier effect on macromolecular drugs, trans‐BBB transport of macromolecules is generally through receptor‐mediated endocytosis (Zhang & Pardridge, 2001). However, under normal circumstances, a small number of lipophile small molecules can directly diffuse across BBB and enter cerebrospinal fluid (CSF) without special conditions (Pardridge, 2015).

The intercellular insertion of TJs into BMECs also provides a protective effect, separating the lumen portion from the basolateral region. Claudins and Occludins are extracellular components of TJs, and key molecules of BBB. TJs is a kind of protein complex mainly formed by the interaction of a variety of transmembrane and cytoskeletal proteins, including claudin‐1/5, occludins, zonula occludens‐1 (ZO‐1), E‐cadherin and actin and plays an important role in biological barriers (Ward et al., 2019; Wolburg & Lippoldt, 2002).

Under physiological conditions, during effective screening of CSF and choroid plexus in BBB, CSF produces small amounts of proteins that are usually retained in BBB without diffusion. BBB damage can be predicted by measuring the levels of these proteins in CSF. These brain‐derived proteins can be used as markers of BBB integrity (Neuwelt et al., 1999). The calcium‐binding protein S100 Beta (S100B) is mainly distributed in ACs and oligodendrocytes, and had high nerve specificity. Neuron specific enolase (NSE) is expressed in neuron cytoplasm, peripheral nerves, endocrine system, platelets and red blood cells, and is mainly concentrated in neurons (Li et al., 2017). Glial fibrillary acidic protein (GFAP) highly expressed in ACs is a kind of skeletal protein with supporting function, supports adjacent neurons and BBB (Zhang, 2021). The three markers can be detected in serum if BBB is damaged (Helmrich et al., 2020). Detection of brain‐derived proteins in serum is considered to be an effective method to evaluate the degree of BBB damage. Different from TJs, brain‐derived proteins can not only reflect the permeability of BBB, but also reflect the damage degree of brain exposed to drug (Helmrich et al., 2020).

Enrofloxacin (ENR) is widely used in animal disease control. Its common products include raw powder, sodium salt, hydrochloride and lactate. However, the antibiotic residue and toxicity to organisms have attracted more attention, such as hepatorenal toxicity (Chen et al., 2019; Zhai & Li, 2017). In aquatic organisms, especially in fish, ENR can accumulate in fish, which impacts growth and development (Chen et al., 2019). Presently, as a member of fluoroquinolones (FQLs), ENR toxicity research mainly focuses on liver, kidney and edible animal tissues including muscle and skin; however, effect of the animal antibiotic on brain tissue or central nervous system (CNS) is still unknown.

Our previous work investigated the toxicity of difloxacin, another member of FQLs, to *Carassius auratus gibelio*, and found that both difloxacin doses, 2480 and 20 mg/kg, could lead to abnormal expression of inhibitory neurotransmitter γ‐aminobutyric acid related anabolic genes, indicating that difloxacin influenced central nervous system (Ruan et al., 2014). ENR has most of the physical and chemical properties of FQLs, such as high lipophilicity, long biological retention and low degradation rate (Wei et al., 2007). So it can be assumed that ENR maybe mediate impact on brain or CNS.

Pengze crucian carp (*Carassius auratus* var. Pengze), an economic species in China, was selected as the experimental object to explore the effects of ENR on the BBB permeability in fish.

## 2 MATERIALS AND METHODS

### 2.1 Experimental animals

The 720 fish (83.60 ± 3.72 g), healthy and free from parasites, were purchased from a farm in Pengze county, Jiangxi Province, and temporarily raised for two weeks with plenty of oxygen, which was pumped into the water. The feeding frequency was twice per day. During the experiment, the aquaculture water was aerated by artificial aerator, and the dissolved oxygen was stabilised at 6.50 ± 0.50 mg/L, and the water temperature and pH were controlled at 25 ± 1°C and 7.50 ± 0.30. Feeding was stopped 24 h before the experiment. Fish were divided randomly into three groups, and 240 fish were placed in each dose group. Each dose group was set into three replicates, that is, each replicate contained 80 fish, control group (fish were orally administrated by normal saline), LD_50_ group (fish were orally administrated by 1949.84 mg/kg ENR) and SD group (fish were orally administrated by 194.98 mg/kg ENR), respectively (Zhao et al., 2013). Fish was severed the connection between spine cord and brain, and placed on ice. Skull was cut open. Intact brain tissue was taken out, immediately cleaned with phosphate buffer solution and then frozen in liquid nitrogen followed by storage at –80°C for usage. The brain tissue and serum of Pengze crucian carp were collected 0.5, 2, 6, 72, 96 and 144 h after administration.

### 2.2 Determination of ENR residue by HPLC

2.0 g brain tissue sample and 12 g anhydrous sodium sulphate were homogenised in 12‐ml acidified acetonitrile with a high‐speed tissue masher. The homogenate sample was placed in a triangular flask with glass beads (2–3 particles/bottle), shook on a shaker for 15 min (120 rpm), centrifuged at 4500 rpm for 15 min. The supernatant was mixed with 30 ml acidified acetonitrile, centrifuged at 4500 rpm for 15 min to harvest supernatant.

10 ml n‐hexane was added into the supernatant and shaken for 5 min. The lower layer of acetonitrile was transferred to the flask and evaporated to dry at 55°C. The residue was fully dissolved with 1.0 ml mobile phase, transferred to a 1.5 ml centrifuge tube and centrifuged at 4500 rpm for 5 min. The supernatant was filtered by 0.45 μm microporous membrane, and the filtrate was subjected to HPLC assay according to the Announcement of Ministry of Agriculture and Rural Affairs of China (No. 783, 2006).

### 2.3 Evans blue staining

After the optimal cutting temperature compound (OCT compound), a water‐soluble mixture of polyethylene glycol and polyvinyl alcohol infiltrates the brain tissue of Pengze crucian carp and the tissue was frozen and embedded on the quick‐freezing table. Brain tissue of Pengze crucian carp was sectioned after the OCT compound turned white and hardens. The brain tissue samples were fixed on the slicer, the tissue surface of the sample was trimmed and then finely cut and the thickness of the section was adjusted to 8–10 μm. After the sections were transferred to a clean slide for labelling, the red evans blue (EB) distribution (EB appears red under green light) was observed under the confocal laser scanning microscope at 405 nm excitation wavelength.

### 2.4 Concentration detection of ENR in brain tissue

The weight of crucian carp brain tissue was accurately weighed. After homogenising the crucian carp brain tissue, 3 ml PBS and 3 ml 60% trichloroacetic acid were added. After fully mixing with a vortex oscillator, the tissue was left standing at room temperature for 30 min. After centrifugation at 3000 r/min for 10 min in a low temperature high‐speed centrifuge, the supernatant was harvested. A series of standard working solution of 200 μl and sample supernatant were added into the 96‐well plate. The absorbance at 632 nm was measured by setting the blank well to zero in the plate.

### 2.5 Quantitative polymerase chain reaction (qPCR)

The primers (*β*‐actin, S100B and GFAP) for qPCR were listed in Table 1, and *β*‐actin was used as the reference gene. The reactions were performed in a thermocycler (Bio‐Rad, USA) with the following profile: one cycle for 30 s at 95°C, followed by 39 cycles for 5 s at 95°C and 30 s at corresponding temperature (Table 1), and 10 s at 95°C, and then 5s at 65°C, then 5s at 95°C. The qPCR was performed in the CFX‐96 real‐time PCR system (Bio‐Rad, USA). The qPCR reactions with 20 μl total volume per reaction (10 μl TB Green Premix, 6.4 μl double distilled water, 0.8 μl of each primer and 2 μl cDNA templates) were set up according to the manufacturer's protocol. Relative gene expression was analysed using the 2^−ΔΔCt^ method.

**TABLE 1:** Information of qPCR primers

Gene	Primer sequences	NCBI accession number	Temperature (°C)
*β*‐actin	F: TACGTTGCCATCCAGGCTGTG R: CATGGGGCAGGGCGTAACC	M_24113.1	55–60
S100B	F: GGGAACCATCATTGAGGTGT R: TGCTCGGTAAGAGTCGGAAA	XM_026235739.1	58.0
GFAP	F: CCTGCTCAATGTCAAACTGGC R: TTCGCACAACTATGCTCCTCTTC	XM_026208575.1	56.5

### 2.6 Western blot

Brain tissue homogenate containing RIPA and PMSF was centrifuged at 4°C, 12,000 rpm for 10 min. Supernatant was determined total protein concentration by BCA method, mixed with loading buffer and then denatured for 10 min by boiling water bath. Samples were subjected to SDS‐PAGE and transferred to PVDF membrane. Following treatment with methanol and skimmed milk powder solution, membrane was probed with primary antibody (antibody NSE: Abcam ab180943, GFAP: Proteintech 60190‐1‐Ig, Occludin: 66378‐1‐Ig, P‐glycoprotein: Rabbit polyclonal antibody, 22336‐1‐AP) and glyceraldehyde‐phosphate dehydrogenase (GAPDH antibody: Proteintech 60004‐1‐Ig, 1:1000). After washing with PBST, membrane was incubated in labelled secondary antibody solution (Proteintech SA00001‐1 or SA00001‐2, 1:500), and then exposed to chemiluminescence fluid in a dark room followed by analysis with Bio‐Rad gel imaging system.

### 2.7 Detection of serum markers of brain injury

According to ELISA manual for experimental operation (MEIMIAN Biological Reagent Co., LTD, Wuhan), serum markers of brain injury were determined.

### 2.8 Data analysis

Data were expressed as mean ± SD, and SPSS 17.0 was used for statistical analysis. One‐way ANOVA and LSD were used to make multiple comparisons for evaluating the statistical significance. *p* < 0.05 was taken as statistical significance.

## 3 RESULTS

### 3.1 ENR can pass BBB in brain crucian carp

**FIGURE 1:**
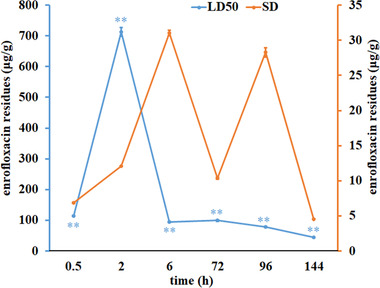
Residual amount of ENR in the brain of crucian carp (*n* = 10). ** expresses *p* < 0.01 between LD_50_ group and SD group

According to the Figure 1, ENR residues in brain tissues from the two treated groups of fish were significantly different and the peak values of ENR were 712.46 ± 14.18 μg/g in LD_50_ group and 31.04 ± 0.36 μg/g in SD group, respectively. In brain tissue of LD_50_‐treated fish, ENR underwent rapid biotransformation, while in brain of SD dose exposed animals, the elimination rate of ENR was slow. The data suggest that ENR can diffuse across BBB into brain tissue.

### 3.2 ENR promotes BBB permeability to EB in crucian carp

**FIGURE 2:**
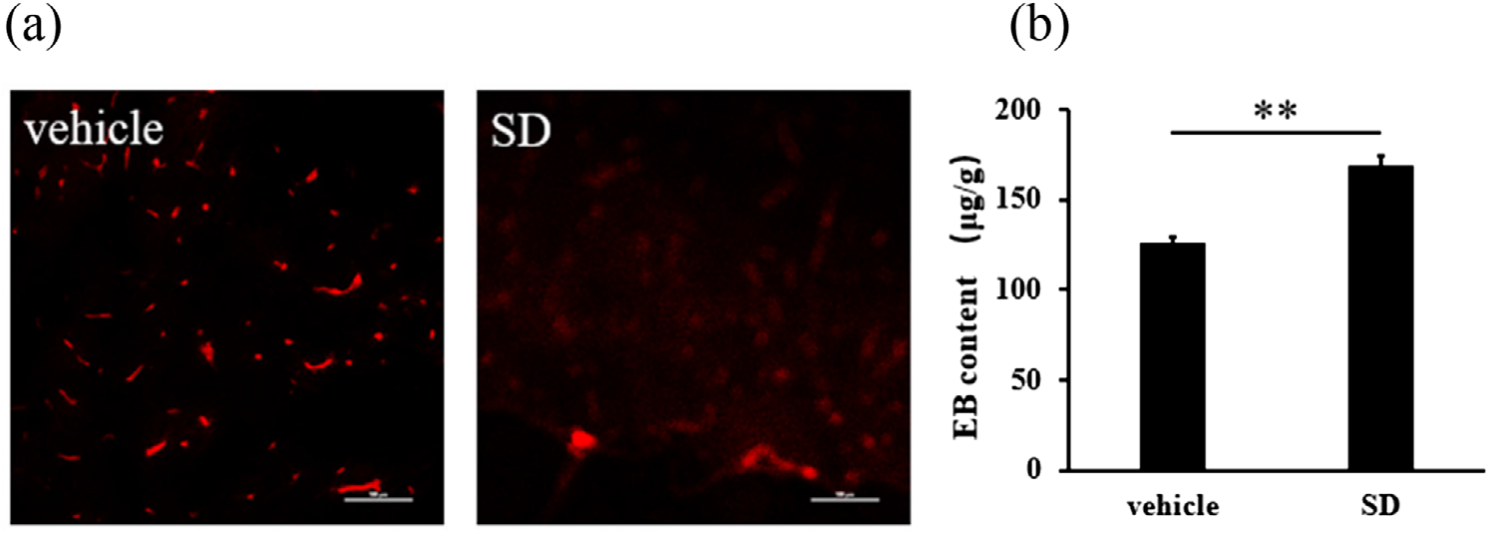
Distribution (a) and content (b) of EB in brain of crucian carp. EB content in the brain of crucian carp after treatment with SD of ENR for 96 h. ** expresses *p* < 0.01

Cerebral slices of crucian carp in the control group showed that EB was clearly visible in brain microvessels (Figure 2), and the contour boundary of vascular was clear, which verified that EB did not penetrate brain microvessels into glial cells. In Figure 2, the permeability of brain microvessels was increased after ENR treatment, and EB was diffused in the brain microvessels of crucian carp, indicating that EB diffused into the glial cells. As shown in Figure 2, after treatment with ENR of SD, EB content in crucian carp brain was significantly increased (*p* < 0.01).

### 3.3 ENR represses expressions of occludin and P‐gp, but promotes expression of GFAP, NSE and S100B in crucian carp brain

**FIGURE 3:**
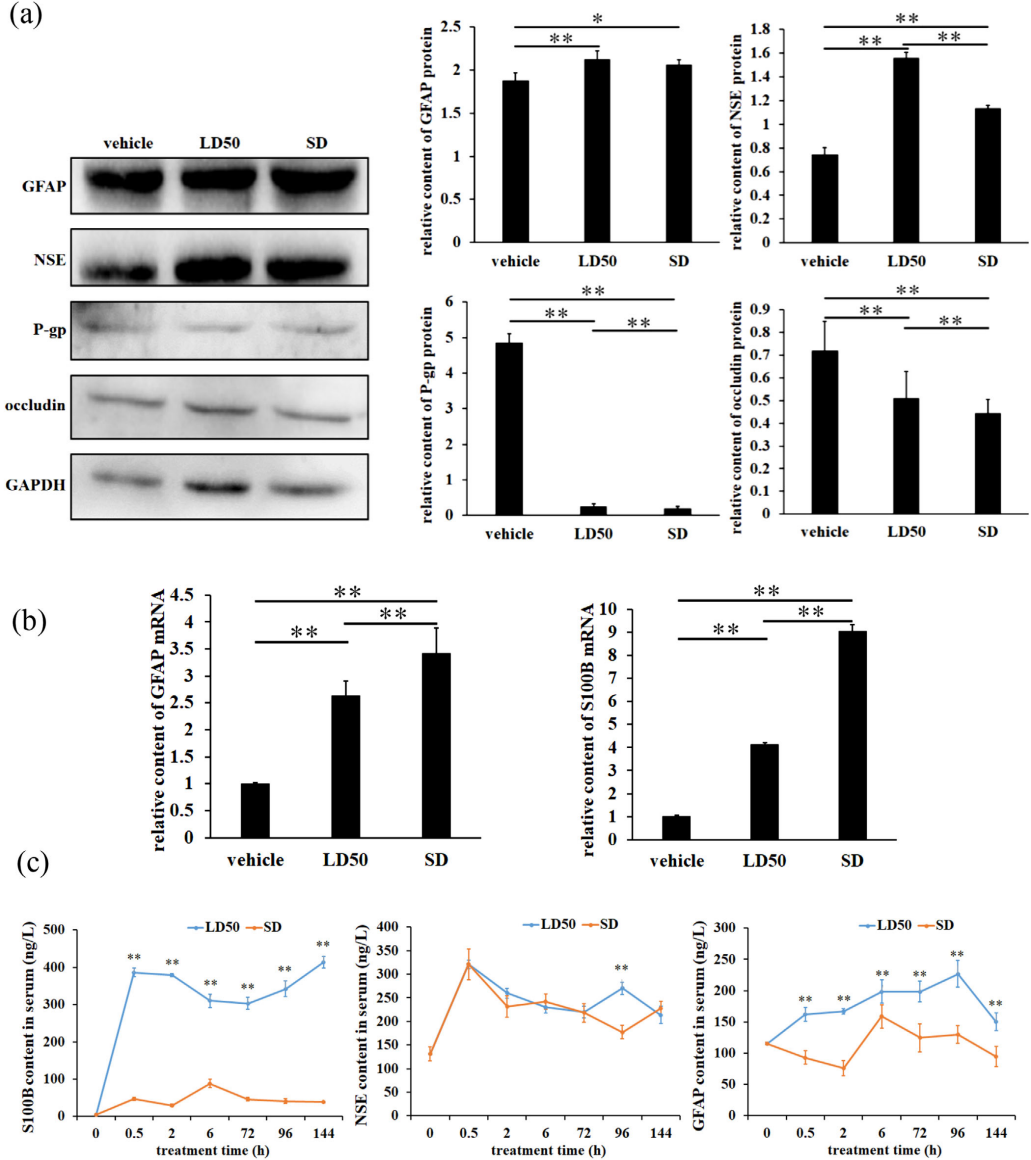
Expressions of some important genes in the brain of crucian carp and contents in serum. (a) BBB‐related protein contents determined by western blot analysis, (b) transcriptional levels assessed by qPCR method and (c) serum contents of brain injury markers determined by ELISA detection kits. * or ** expresses *p* < 0.05 or *p* < 0.01, respectively

Data in Figure 3a present that contents of crucian carp BBB related proteins, occludin and P‐gp, were significantly downregulated by single oral administration of ENR at LD_50_ and SD (*p* < 0.01); moreover, LD_50_ ENR was observed more obvious inhibition than SD ENR (*p* < 0.01). Meanwhile, ENR exposure elevated protein contents of GFAP and NSE in animal brain (*p* < 0.01 or *p* < 0.05), LD50 ENR was more effective in upregulating expression of NSE than SD ENR, and significant difference between LD50 treated and SD treated fish was observed in NSE (*p* < 0.01), not in GFAP (*p* > 0.05). Apart from augment of protein content, ENR‐induced expression of GFAP was supported by increased transcript product (*p* < 0.01, Figure 3b).

Detection of transcript products also revealed that S100B was promoted by ENR (*p* < 0.01, Figure 3b), the translation level could be reflected by serum content of S100B, and markedly high S100B protein content was observed after treatment with ENR (*p* < 0.01, Figure 3c). Data in Figure 3c also showed that ENR increased NSE and GFAP protein contents in serum. LD_50_ ENF caused extremely significant increase of GFAP protein content in serum of crucian carp (*p* < 0.01), but the change of GFAP protein content in SD group was unstable, and the difference of GFAP protein content between the two groups was extremely significant (*p* < 0.01).

### 3.4 Correlation analysis between ENR residues and serum S100B, NSE and GFAP

Data in Table 2 present that, according to the correlation between ENR and the contents of three serum brain injury markers analysed by Spearman rho in SPSS, the correlations between ENR and S100B, NSE and GFAP were 0.853 (*p* < 0.01), 0.140 and 0.657 (*p* < 0.05), but no close correlation was observed between ENR residue and NSE (*p >* 0.05).

**TABLE 2:** Correlations between ENR residues and serum S100B, NSE and GFAP

Item	Correlation coefficient	Significant level
ENR residues/S100B	0.85	*p* < 0.01
ENR residues/NSE	0.14	*p >* 0.05
ENR residues/GFAP	0.66	*p* < 0.05

## 4 DISCUSSION

The barrier permeability of FQLs has been reported for a long time. Escudero and co‐workers detected the presence of difloxacin in goat milk, demonstrating the ability of difloxacin to penetrate the haemothorax barrier into the udder (Escudero et al., 2011). It is reported that corresponding drugs can be detected in the brain tissue of rats after FQLs perfusion (Jaehde et al., 1993). In this work, ENR residue was detected in brain tissue of crucian carp exposed to ENR, two doses of LD_50_ and SD, peak values of ENR residue were 712.46 μg/g and 31.04 μg/g in LD_50_ group and SD group, respectively. As a member of FQLs, ENR could stay in the brain tissue of crucian carp in large quantities, indicating that ENR possesses high BBB permeability.

Structurally, permeability of BBB is mainly controlled by BMECs, TJs and matrix membranes; it is difficult for substances to penetrate through the intact BBB into brain tissue. BBB permeability is easily affected by a variety of factors. For instance, unconventional means, such as heat stress, noise and ultrasound, can increase BBB permeability of mice (Ohta et al., 2020; Sun et al., 2021). Chen et al. (2020) found that intraperitoneally injected muskone could change the ultrastructure of BBB in zebrafish. FQLs have high lipid solubility, so they can penetrate BBB and enter the brain easily (Ruan et al., 2014). According to the findings of this work, ENR exposure promoted EB diffusion, indicating that ENR improved permeability of BBB, then resulted in EB crossing BBB. This result further confirms that ENR can cross BBB via changing the permeability of BBB. Consequently, ENR penetrates BBB of the fish via both lipid diffusion and changing BBB permeability.

BBB permeability is regulated by a variety of factors, such as protein modification site changes (Goncalves et al., 2013), temperature (Uchida et al., 2019), heavy metals (Wang et al., 2017) and drugs (Chen et al., 2020), which can affect the structure and function of BBB. Occludin is an important molecules of tight junction widely distributed in body barrier. As an important gene in TJs, occludin is highly expressed in BBB and plays key role in the regulation of BBB function (van Leeuwen et al., 2018; Zhang et al., 2010). P‐gp, a transporter of BBB, plays an important role in maintaining stability of internal environment of brain (Ding et al., 2021). In this study, it was found that expression of occludin in TJs of crucian carp BBB was extremely significantly downregulated by ENR. This result suggested that the change of BBB permeability to ENR might be mediated by inhibiting expression of occludin in crucian carp brain.

P‐gp is an important transporter of BBB. Clinically, P‐gp inhibitors are used to extend retention time of drugs in brain (Nobili et al., 2012). This work revealed that ENR not only reduced the expression of TJs‐related gene occluding but also significantly inhibited P‐glycoprotein, resulting in more ENR existed in brain tissue of Pengze crucian carp and consequent brain injury. S100B, NSE and GFAP have been recognised as marker molecules for astrocytes and neurons in brain, so their expression levels reflected the degree of brain injury. Our findings showed that ENR improved expression of S100B, NSE and GFAP in crucian carp brain, and higher contents of them were observed in serum. This result suggests that ACs are in a state of activation. ACs can be regulated by some drugs. Resveratrol is beneficial for brain injury caused by oxygen and sugar deprivation via inhibiting expression of S100B and GFAP and consequent activation inhibition of ACs (Liu et al., 2020). Sevoflurane can induce expression of GFAP and activation of ACs and, resulting in neurological disorders in mice (Zhang, 2020).

In the present study, we first revealed that ENR improved BBB permeability of crucian carp by mediating structure disruption, and consequently more ENR entered brain tissue through BBB. Elevated ENR‐residue‐induced brain injury was related to activation of ACs and neuron injury.

## AUTHOR CONTRIBUTIONS

Fan Zhang: investigation; methodology; writing – original draft. Runping Wang: investigation. Jianzhen Huang: methodology; project administration. Jiming Ruan: data curation; funding acquisition; supervision. Huazhong Liu: conceptualisation; supervision; writing – review & editing.

## CONFLICT OF INTEREST

The authors declare no conflict of interest.

### ETHICAL APPROVAL

The entire experimental procedure was approved by the Animal Care Commission of the College of Animal Science and Technology, Jiangxi Agricultural University, China.

### PEER REVIEW

The peer review history for this article is available at https://publons.com/publon/10.1002/vms3.918.

## Data Availability

Data sharing is not applicable to this article as no new data were created or analyzed in this study.
